# Reflecting on shared decision making: A reflection‐quantification study

**DOI:** 10.1111/hex.12953

**Published:** 2019-08-14

**Authors:** Marleen Kunneman, Christina M. LaVecchia, Naykky Singh Ospina, Abd Moain Abu Dabrh, Emma M. Behnken, Patrick Wilson, Megan E. Branda, Ian G. Hargraves, Kathleen J. Yost, Richard M. Frankel, Victor M. Montori

**Affiliations:** ^1^ Knowledge and Evaluation Research (KER) Unit Mayo Clinic Rochester Minnesota; ^2^ Medical Decision Making, Department of Biomedical Data Sciences Leiden University Medical Center Leiden The Netherlands; ^3^ Division of Endocrinology, Department of Medicine University of Florida Gainesville Florida; ^4^ Department of Family Medicine Mayo Clinic Jacksonville Florida; ^5^ Division of Health Care Policy and Research, Department of Health Sciences Research Mayo Clinic College of Medicine Rochester Minnesota; ^6^ Indiana University School of Medicine Indianapolis Indiana; ^7^ Education Institute Cleveland Clinic Cleveland Ohio

**Keywords:** argumentation, communication, measurement, patient involvement, shared decision making

## Abstract

**Background:**

Reflecting (“stop‐and‐think”) before rating may help patients consider the quality of shared decision making (SDM) and mitigate ceiling/halo effects that limit the performance of self‐reported SDM measures.

**Methods:**

We asked a diverse patient sample from the United States to reflect on their care before completing the 3‐item CollaboRATE SDM measure. Study 1 focused on rephrasing CollaboRATE items to promote reflection before each item. Study 2 used 5 open‐ended questions (about what went well and what could be improved upon, signs that the clinician understood the patient's situation, how the situation will be addressed, and why this treatment plan makes sense) to invite reflection before using the whole scale. A linear analogue scale assessed the extent to which the plan of care made sense to the patient.

**Results:**

In Study 1, 107 participants completed surveys (84% response rate), 43 (40%) rated a clinical decision of which 27 (63%) after responding to reflection questions. Adding reflection lowered CollaboRATE scores (“less” SDM) and reduced the proportion of patients giving maximum (ceiling) scores (not statistically significant). In Study 2, 103 of 212 responders (49%) fully completed the version containing reflection questions. Reflection did not significantly change the distribution of CollaboRATE scores or of top scores. Participants indicated high scores on the sense of their care plan (mean 9.7 out of 10, SD 0.79). This rating was weakly correlated with total CollaboRATE scores (rho = .4, *P* = .0001).

**Conclusion:**

Reflection‐before‐quantification interventions may not improve the performance of patient‐reported measures of SDM with substantial ceiling/halo effects.

## 1 INTRODUCTION

In shared decision making (SDM), patients and clinicians work together in making decisions about health and care.^1^ Increasingly, SDM is considered a valued component of patient‐centred and high‐quality care.^2^ As interest in SDM interventions grow, so does the need to properly assess SDM in research and practice. However, measurement challenges make it difficult to evaluate the occurrence of SDM.

Specifically, measurement of SDM is limited by currently available assessment instruments. A recent systematic review by Gärtner et al identified 40 SDM measurement instruments.^3^ Most were developed for third‐party evaluation, requiring resources and time to observe and code SDM, which hinders their application in large‐scale assessment. Other SDM instruments ask patients or clinicians to self‐report the perceived degree of SDM. These brief, self‐reported instruments have caught the attention of funders and policy makers for evaluating SDM on a large scale due to their ease of use and efficient administration. These instruments, however, have potential pitfalls. Previous research has shown discrepancies between observer evaluations and self‐reported perceptions of SDM.^4^ Patient‐reported SDM scores are usually higher and tend to have substantial ceiling effects: scores are maximum without much variance.^4^, ^5^ This may be due to a lack of patient familiarity with SDM or of training in its evaluation, such that patients may have difficulties disentangling the evaluation of SDM from the evaluation of other aspects of care or from their overall satisfaction (halo effects).^6^ These ceiling and halo effects limit the responsiveness of self‐reported SDM assessments in individual encounters and require larger groups of patients to detect differences in SDM performance across clinicians and clinics.

In addition, SDM instruments seem to have a strong focus on process: on *what* is done or which ‘technical’ steps are taken.^7^ There is much less attention on *how* these steps are taken—for example, whether a humanistic approach was used (respecting the patient's humanity and acting with compassion, integrity, and empathy in both the manner and content of the interaction),^7^, ^8^, ^9^ or on the extent to which the resulting decision makes sense: that is, patients and clinicians *know and understand* that the decision made is the best way forward and that it also *feels right* and *can be implemented* in the lifeworld of the patient.^1^

We tested whether the occurrence of SDM could be assessed better by using a reflection‐quantification rubric: eliciting patient reflections before requesting a numerical evaluation. We hypothesized that reflection could introduce a pause (“stop‐and‐think”) when using self‐reported brief SDM instruments, slowing patients down and encouraging them to reflect above and beyond their assessment of general satisfaction with the clinician or the visit.^5^, ^10^, ^11^ Also, written reflections may reveal *why* patients value the SDM process, which makes words (rather than numerical ratings) ‘peculiarly appropriate for judging quality within healthcare’.^12^ To address this possibility, we set up two studies to assess (a) the extent to which reflective questions can improve the responsiveness of patient‐reported SDM evaluations of individual encounters and (b) the concordance between patients’ evaluations of the SDM process and of the extent to which the resulting plan of care makes sense to them.

## 2 METHODS

This paper reports on two studies that used two different approaches to add reflective questions to quantitative SDM evaluations. In the second study, we also added items on how much sense the decided‐upon plan of care made to patients. These studies are part of the Fostering Fit by Recognizing Opportunity STudy (FROST) programme. This programme of work focuses on understanding and advancing care that fits the lives of patients. The Mayo Clinic Institutional Review Board (IRB No. 16‐010422) approved both studies.

## 3 STUDY 1. REPHRASING ITEMS INTO REFLECTION QUESTIONS

### 3.1 Study 1: Methods

#### 3.1.1 Study population

Consecutive adult patients and their companions, without exclusions, visiting the Division of Endocrinology at the Mayo Clinic (Rochester, MN) outpatient area for a scheduled appointment were eligible for the study.

#### 3.1.2 Questionnaires

The basis of our experimental approach is to use CollaboRATE, a widely used SDM self‐reported instrument,^5^ as the starting questionnaire. This instrument was designed to rate clinicians or clinics efficiently.^13^ Results are reported as the percentage of patients visiting a clinician or a clinic who rated all three CollaboRATE questions using 9, the highest rating possible.

In addition to CollaboRATE (version 1), we developed two other versions in which each item was preceded by a reflective question with a similar wording, structure and focus. Version 2 asked patients to think of a particular visit, and version 3 of a particular decision (Appendix S1). For example, CollaboRATE asks patients, “How much effort was made to help you understand your health issues?” Versions 2 and 3 ask this question after asking, “Which efforts were made to help you understand your health issues? ”

All three versions collected age, gender and level of schooling but no identifiable participant information. The triage question “Can you remember a recent clinical appointment in which an important health decision was made (within the last month)? ” followed. Those who remembered went on to complete the rest of the questionnaire.

#### 3.1.3 Procedure

At check‐in for their outpatient appointment, patients and, if present, their companions were asked to participate. Surveys had a cover sheet that blinded coordinators to the version of the survey and were numbered, using simple randomization, for order of distribution. Patients were asked to return the questionnaire to the clinic receptionist before leaving the outpatient area, either empty (indicating no participation) or filled in (indicating participation).

#### 3.1.4 Statistical analyses

Although we sought to evaluate the performance of reflection questions to obtain preliminary estimates of effects, nonetheless we compared outcomes using the Fisher exact test for categorical variables and the Wilcoxon rank sum test (two‐sample comparisons) or Kruskal–Wallis test (three‐sample comparisons) for continuous variables. To visualize the association between variables, we characterized the entire distribution using smoothed density estimates. All analyses were performed by using R version 3.2.3 software^14^ and SAS 9.4. Statistical comparisons were two‐sided and were considered significant at the *P* < .05 level. The content of the reflection questions was not analysed for the purpose of this study.

### 3.2 Study 1: Results

#### 3.2.1 Participants

Over the course of two days in April 2017, we distributed 127 and received 107 completed questionnaires (84% response rate). Of these participants, 43 (40%) remembered a clinical appointment in which a decision was made, thus becoming eligible for, and completing, the rest of the questionnaire. Of these 43, 16 (37%) filled in version 1 (unchanged CollaboRATE), 14 (32%) version 2 (reflection on visit + CollaboRATE) and 13 (30%) version 3 (reflection on decision + CollaboRATE) of the questionnaire. Participants’ mean age was 53 (SD 16), 14 (33%) were women, and 7 (16%) had a high school education or less; there were no differences in these characteristics between the three groups.

#### 3.2.2 Reflection‐quantification

**Figure 1:**
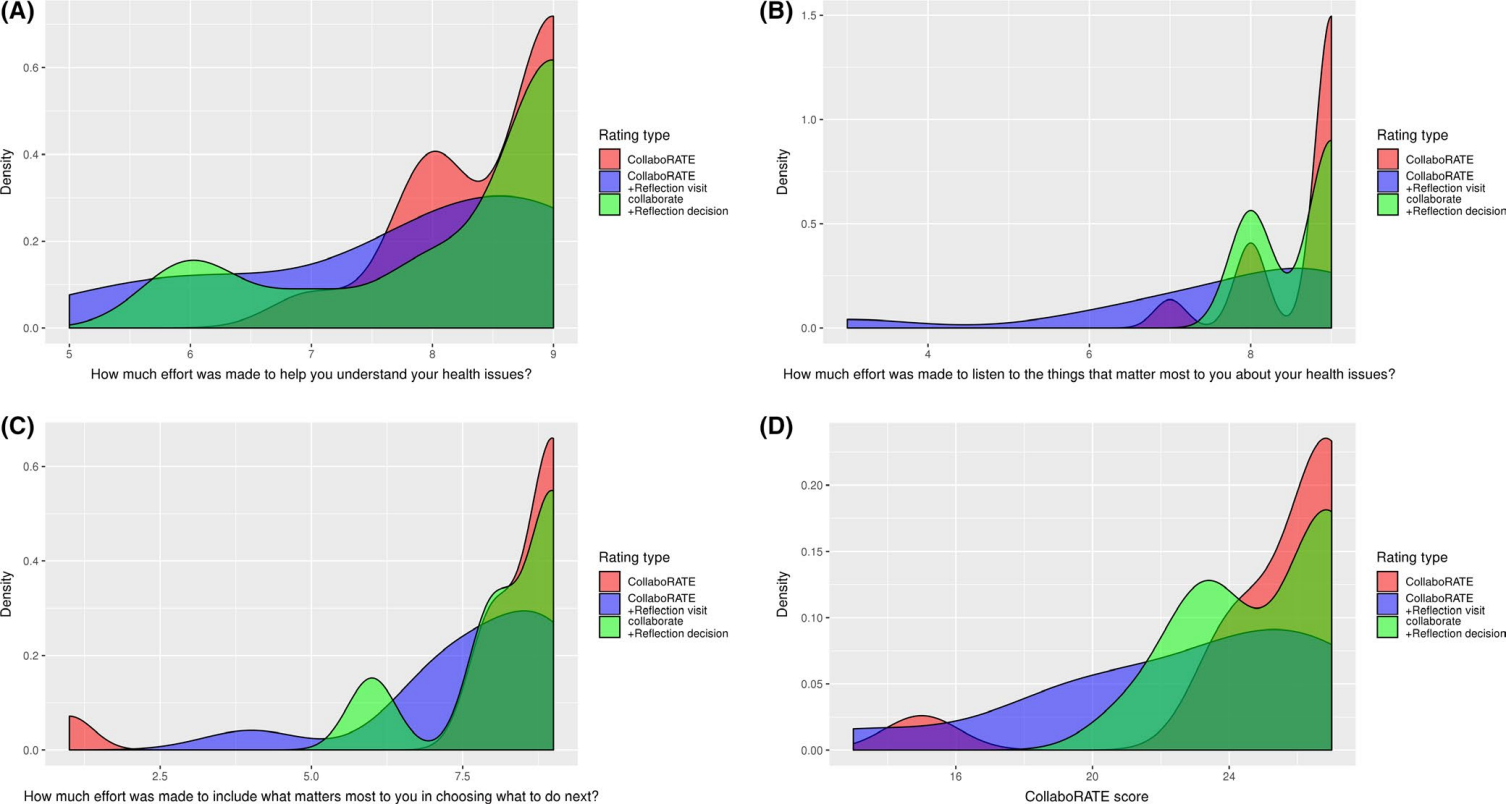
(A‐D) Density estimates for CollaboRATE total score and for each individual item by rating type

Adding reflection questions to CollaboRATE reduced the proportion of patients reporting the maximum score (N = 10/16, 63% for version 1; N = 5/14, 36% for version 2; and N = 6/13, 46% for version 3). Similarly, reflection questions led to lower scores for individual CollaboRATE items and for the sum score (Figure 1A‐D). Although some of these differences seem large and potentially important, all of these differences were not statistically significant.

## 4 STUDY 2. REFLECTING ON THE CONVERSATION AND ON HOW MUCH SENSE THE DECIDED‐UPON PLAN OF CARE MAKES

### 4.1 Study 2: Methods

#### 4.1.1 Study population

Consecutive adult patients at the outpatient clinics of the Department of Endocrinology of the University of Florida (Gainesville, FL), and the Departments of Family Medicine and Primary Care Internal Medicine of Mayo Clinic Florida (Jacksonville, FL), without exclusions, were eligible for the study. A priori sample size estimations called for recruiting 200 participants (100 per site) to detect a minimum 5% difference between versions in total CollaboRATE score using a standard deviation 2.7 with 90% power and two‐sided alpha of 5%. The difference is not reflective of a meaningful difference, but instead represents the difference detectable based off sample size.

#### 4.1.2 Questionnaires

As in Study 1, we used CollaboRATE as the starting instrument. Apropos of its three questions, we built reflection questions. The process we followed to determine the questions and refine them to feasibly improve their ability to promote deep reflection required three rounds of think‐aloud interviews with patients attending the Mayo Clinic (Rochester, MN).

In the first round, we started with the reflection questionnaire used in Study 1. A researcher (MK) observed clinical encounters with four patients with diabetes at the outpatient endocrinology clinic. After their encounters, she asked them to read the questions and think aloud while verbalizing their responses. Patients indicated they were confused about the questions, mainly by the term “efforts”. They mostly reflected on characteristics of the clinician (“He is always so nice”). Also, after filling in the first quantitative score, patients seemed to anticipate the next score and used it to justify the score they knew they were going to give.

To correct this, we formulated new reflection questions (see final version, questions 1, 3 and 4) and placed all these questions at the start of the questionnaire, before any of the CollaboRATE items. A researcher (MK) observed the clinical encounters of six patients at either an outpatient endocrinology clinic or at a family medicine clinic, and after their encounter asked patients to read the reflection questions and think aloud while writing down their responses. We observed a delay between reading the reflection questions and answering them, and patients also paused while writing their answers down.

In the third round, we added a reflection question asking patients to describe aspects of the conversation with the clinician that did not go well (see final version, question 2) and we added a linear analogue self‐assessment scale on how much sense the decision made to them. A researcher and clinician (VMM) observed encounters of four patients at the endocrinology clinic and asked them after their encounter to read the questions and think aloud while writing down their responses. Patients reported understanding the reflection questions. They often compared the conversation they had just had with previous encounters (either with the same or a different clinicians). In arguing for how sensible the decision was to them, they mostly offered intellectual (eg “the rationale behind it was easy to understand”) and emotional (eg “this feels right”) arguments.

For Study 2, we used CollaboRATE (version 1) or a reflection‐quantification questionnaire (version 2). Both versions collected demographics (without participant identifiers) and the health problem motivating the visit. Version 2 asked four reflection questions: 1. What about the conversation went well? 2. What about the conversation could be improved? 3. Do you think that your clinician understands you and your situation? Tell us why you think that. 4. How are you and your clinician dealing with your situation? Tell us what you are planning to do. These were followed by a reflection question on sense (‘Why does that plan make sense to you?’), and a linear analogue self‐assessment (LASA, 0‐10) scale on sense (Appendix S2).

#### 4.1.3 Procedures

We asked patients to participate when they checked in for their appointment. Survey randomization was the same method as Study 1. Patients received one of the two questionnaire versions and were asked to complete them immediately after their encounter and to return them before leaving the outpatient area.

#### 4.1.4 Response classification

Two researchers (MK and VMM) working together categorized the nature of the health issue motivating the visit into acute (a current complaint), chronic (care for an ongoing condition) or preventive (care to avoid a condition). Additionally, two researchers (MK and CML), working together until reaching consensus, categorized the reasons participants gave for why the decided‐upon plan of care made sense into intellectual (participants could understand the plan's justification), emotional (participants feel favourably towards the plan) or practical reasons (participants consider the plan workable).

#### 4.1.5 Statistical analyses

Outcomes were compared using the Fisher exact test for categorical variables and the Wilcoxon rank sum test for continuous variables. The Brown‐Forsythe test was used to test for the equality of group variances. To assess correlations between variables, we used Spearman's rank correlation. To visualize association between variables, we implemented simple scatter plots as well as smoothed density estimates. All missing data were handled using complete case analyses. All analyses were performed using R version 3.2.3 software and SAS 9.4. Statistical comparisons were two‐sided and were considered significant at the *P* < .05 level.

### 4.2 Study 2: Results

#### 4.2.1 Participants

The survey was administered between February and July 2018. Table 1 describes participant characteristics. A total of 109 patients (51%) filled in version 1 (CollaboRATE), and 103 patients (49%) filled in version 2 (reflection + CollaboRATE).

**Table 1:** Participant characteristics in Study 2 (N = 212)^a^

Patient characteristics	N (%)
Age, mean (SD)	54 (17)
Gender, female	134 (64%)
Education	
High school graduation or less	25 (12%)
Some college or college graduation	130 (62%)
Graduate or professional school degree	53 (25%)
Location	
Primary care—Mayo Clinic—Jacksonville, FL	116 (55%)
Endocrinology—University of Florida—Gainesville, FL	96 (45%)
Health issue	
Acute concerns	35 (17%)
Chronic/ongoing concerns	149 (70%)
Preventive care	28 (13%)

a There were no significant differences between study arms across all listed characteristics.

#### 4.2.2 Reflection‐quantification

**Figure 2:**
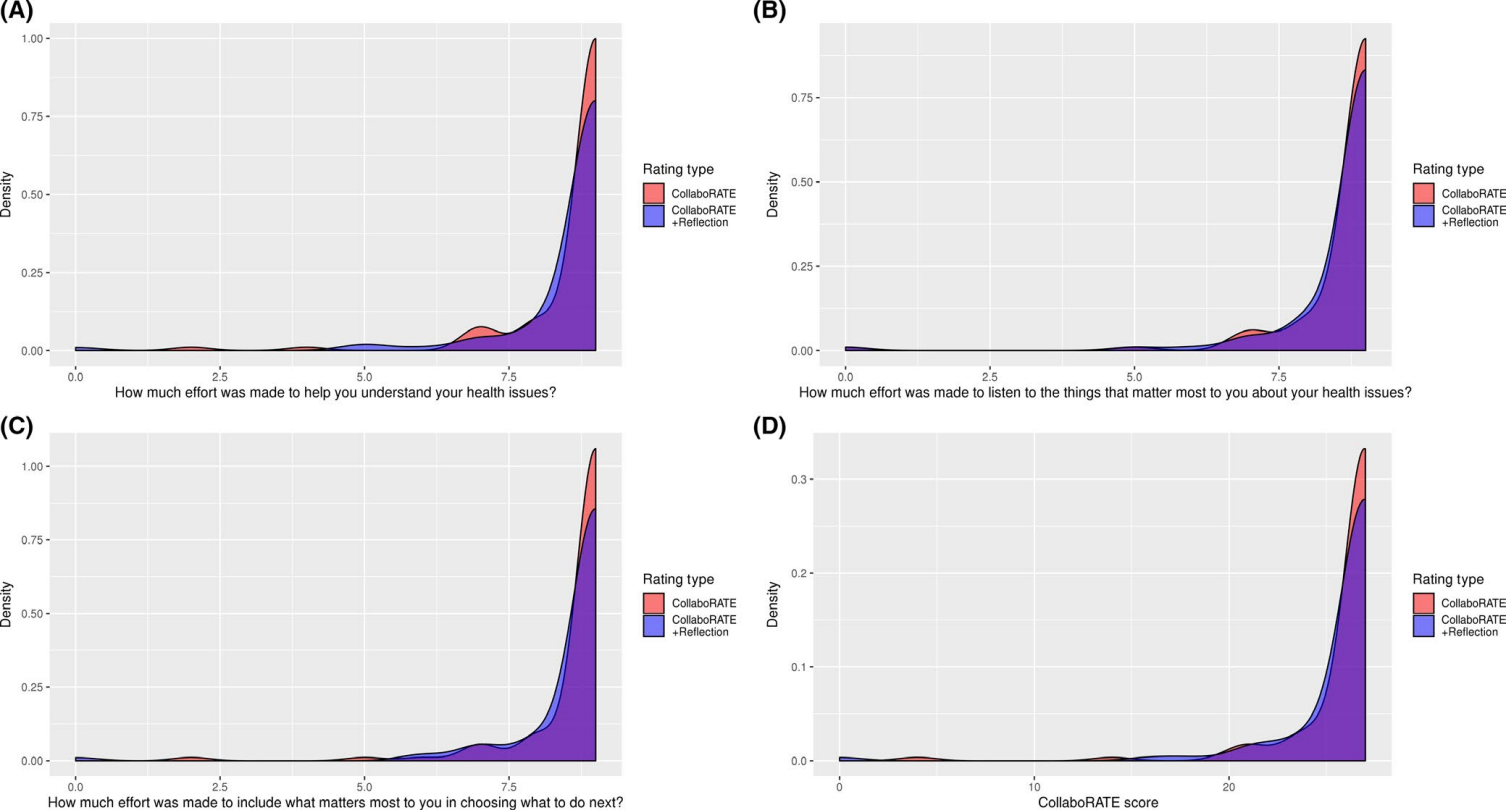
(A‐D) Density estimates for CollaboRATE total score and for each individual item by rating type

Adding the reflection questions lowered individual CollaboRATE item scores and the total score (shifting results to the left as shown in Figure 2A‐D), but did not significantly change the distribution of patients reporting the maximum CollaboRATE score (version 1, N = 87/109, 81% vs. version 2, N = 77/103, 82%) or the mean score and variance of either individual CollaboRATE items or total CollaboRATE scores. These results were robust to the exclusion of patients who did not respond to the reflection questions (N = 18).

#### 4.2.3 What about the conversation went well? What about the conversation could be improved?

When reflecting on what about the conversation went well, about a third of participants gave a cursory response that everything had gone well (eg “All of it”, “Everything”, “Yes”) or returned a blank response. Others commented on the clinician's persona, for example “Honest and very firm”, “She is very knowledgeable, thorough, and patient” or that the clinician “cared” or was “attentive”. About half directly assessed their care or the communication process itself, for example “The doctor described and showed me pictures. Very informative”; “[Clinician name] communicated her diagnosis well”; “He cared and listened. He developed an action plan”; “Listened to me, took me seriously, was encouraging”; “She listened!! Didn't rush me, looked at me as a total person not an organ system, educated me”; “[Clinician name] listened to my needs and adjusted how I would be treated today”.

Most participants stated nothing about the conversation could be improved (“Nothing”, “None” or “N/A”), left the question blank or simply repeated that everything had gone well (eg “Nothing, I left with a total understanding of the diagnosis”). Only four responses noted complaints; of these, three could be interpreted as having direct impact on the nature of SDM. Two participants noted that time was a concern (“Wait time” and “More time”) and another participant was dissatisfied with the clinician they saw, saying “3rd year med student not super equipped to troubleshoot ankle pain in high level athlete”. Last, a fourth participant seemed to indicate that the situation itself was a source of dissatisfaction, saying “Wish there was a magic pill”.

#### 4.2.4 How sensible is the decided‐upon plan of care?

**Figure 3:**
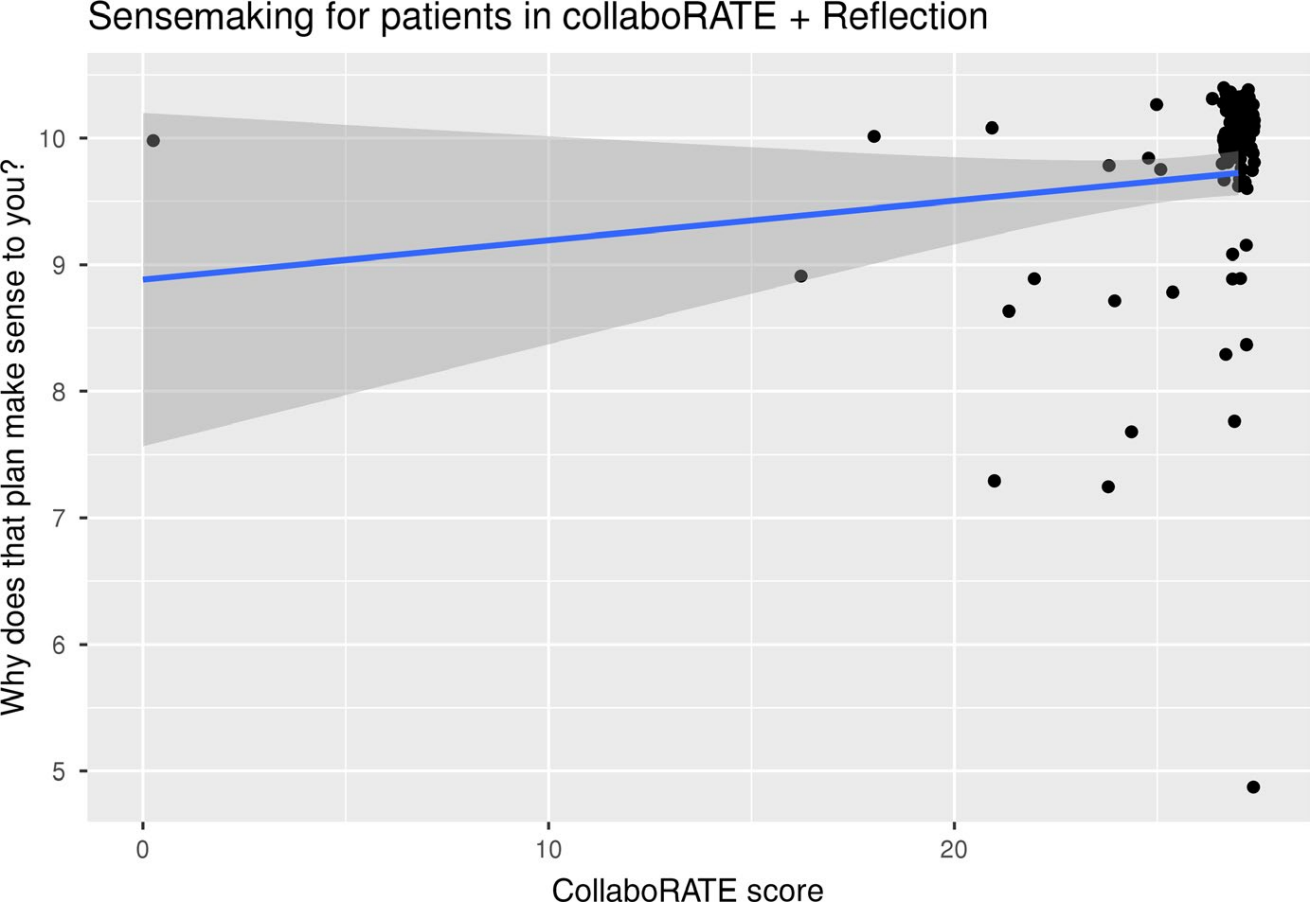
Scatterplot of CollaboRATE scores and ratings of decision sense

Of 103 patients, 82 (80%) offered arguments for why their care plan made sense. Patient most often indicated intellectual arguments for why their care plan made sense (50/82, 61%), with fewer patients indicating their plan made emotional (29/82, 35%) or practical sense (3/82, 4%) to them. Table 2 lists some typical examples of the arguments they offered. LASA scores on sense were highly skewed towards the maximum score of 10 (mean: 9.7; SD: 0.79). Sense scores were significantly (*P* = .0001), albeit weakly (rho = .4), correlated to total CollaboRATE scores (Figure 3) and were not related to whether patients reported intellectual versus other forms of sensemaking (data not shown).

**Table 2:** Common arguments patients used to justify how much sense the decision taken made to them^a^

**Intellectual sense**
We used up‐to‐date knowledge and current recommendations.
We agreed about doing the tests.
The plan was fully explained in terms I could understand.
Laboratories need to be completed to find out the next steps for treatment.
**Emotional sense**
It is what I was told would happen.
I believe in my doctor's advice. I believe in him.
I have a comfortable feeling.
After 10 y, I am ready to see if this is the answer.
**Practical sense**
I will start [new medication] if cost with my insurance is not too high. If it is, I will remain on [current medication].
[The plan] seems easy to follow

a Participants’ reasons were categorized into intellectual (participants could understand the plan's justification), emotional (participants feel favourably towards the plan) or practical sense (participants consider the plan workable).

## 5 DISCUSSION

Introducing a pause for participants to reflect before responding to each item did not affect the distribution of CollaboRATE responses. We have not been able to find precedents for the use of “stop‐and‐think” reflection items to improve the performance of self‐reported patient experience measures per se. At least three arguments have suggested reflection‐quantification could work. Survey researchers have recognized “order effects”, whereby answering preceding questions can affect how participants answer subsequent ones.^15^ Here, reflection questions, via this so‐called order effect, could prime the responder and anchor their views about the specific SDM experience being evaluated, which should have increased variance in CollaboRATE responses across patients with different SDM experiences. Alternatively, preceding questions could, in theory, help with the recall of specific SDM experiences from memory and make them more available for quantification. Quantification, in turn, would then reflect judgements on that particular SDM conversation rather than on the whole encounter, the ongoing relationship with the clinician or clinician attributes, reducing halo effects. Finally, according to dual‐process theory of reasoning,^16^ the System 1 process—often characterized as fast, automatic and subconscious reasoning—may engage satisficing in which the respondent answers the 3‐item questionnaire, but does not do so thoughtfully.^17^ The response patterns may present itself as straight lining by which participants choose the maximum response for all questions. The request for reflection might instead elicit judgements from System 2 process; the slow, conscious, deliberate and analytical reasoning process.

Despite these possibilities, the approaches we tested to promote a reflective pause prior to quantification, introducing reflection before the scale or before each individual item, had minimal if any effects on CollaboRATE ratings. Perhaps, our efforts to induce a ‘stop‐and‐think’ pause before rating were inadequate (i.e., responders needed more help to evaluate the SDM work they did) or insufficient (i.e., they did not draw sufficient attention to the experience). Alternatively, and as suggested by some participant reflections, patients may have experienced what they considered high‐quality SDM or high‐quality care more generally, that is, their reported scores accurately reflected their experience and were not affected by measurement bias such as satisficing at ceiling or halo effects.

Our findings—some important differences in Study 1, no significant differences with reflection in both studies—may inspire further exploration of reflection‐before‐quantification manoeuvres. Yet, future efforts may need to consider other ways to induce a reflective pause and test it in situations in which the quality of SDM is known to be lower or more variable. Recording encounters to ascertain the quality of the SDM process through a third‐party measure (e.g., the OPTION scale^18^) may help interpret questionnaire results in future studies. Review of recordings with patients may elicit judgements that could be used to develop new reflection‐before‐quantification prompts. Alternatively, CollaboRATE may simply lack sufficient reliability at the upper scale range to discriminate discrete levels of SDM in individual encounters, and it may be worth exploring how “stop‐and‐think” approaches affect measures capable of producing more variable ratings, for example SDMQ9.

In summary, the methods used have some limitations. We included a relatively small sample of patients from only three sites in the United States, limiting the generalizability of our study. The participants in our pilot study were encouraged to think aloud, but other participants only reflected “on paper”, which may have been inadequate or insufficient. In addition, we only used one SDM measurement instrument. Perhaps, other instruments would have been more sensitive to the influence of reflection.

Although the open‐ended patient reflections did not change score distributions, they point towards the methodological difficulty of capturing substantial assessments of the nature of SDM conversations in these encounters. For example, while most participant reflections expressed satisfaction with their SDM encounters, only a small number identified aspects of the encounter directly pertaining to SDM. Most of the remaining responses indicated that patients’ satisfaction primarily lay with their sense of who their clinician is (e.g., someone “caring” or “attentive” or “understanding”) or lacked substance (eg responding “Everything” or “All of it” went well in the encounter), implying that participants’ scores were more a function of a social expectation than of an authentic evaluation of the SDM in the encounter.^19^ Yet, these same patients gave those same experiences maximum CollaboRATE scores. This finding should give pause to those calling for the widespread adoption of this measure in quality improvement and SDM implementation efforts. Alternatively, it is conceivable that scoring high on the technical steps of SDM, as measured using SDMQ9 for example, may not necessarily lead to a decision that makes sense and vice versa, a possibility that those seeking to improve SDM measures may want to explore further.

In conclusion, we found limited and somewhat inconsistent evidence that reflection‐before‐quantification interventions may improve the performance of patient‐reported measures of SDM with substantial ceiling and halo effects. Future steps need to consider other ways to induce a reflective pause and test it in situations in which the quality of SDM is known to be lower or more variable.

## CONFLICT OF INTERESTS

The authors declare that there is no conflict of interest.

## Data Availability

The data that support the findings of this study are available from the corresponding author upon reasonable request.
