# Supplementary material for: Reflecting on shared decision making: A reflection‐quantification study
Source: Health Expect. 2019 Aug 14;22(5):1165–72. doi: 10.1111/hex.12953 (PMC6803557; doi:10.1111/hex.12953)
Supplement: Supplementary file 1 [file HEX-22-1165-s001.docx]

**Appendix 1**. **Reflection questions used in Study 1**

**Appendix 2**. **Reflection and sense questions used in Study 2**
